# Patterns of Care in the Final Week of Life: A Comparative Analysis of Aggressive Treatment Versus Comfort-Centered Care Across Healthcare Settings

**DOI:** 10.3390/medicina62091793

**Published:** 2026-09-17

**Authors:** Camelia Ancuta, Nicoleta Mitrea, Mariana Sporis, Flavia Hurducas, Daniela Mosoiu

**Affiliations:** 1 Department of Fundamental Disciplines and Clinical Prevention, Faculty of Medicine, University of Transilvania, 500036 Brașov, Romania; nicoleta.mitrea@hospice.ro; 2 Hospice Casa Sperantei, 500074 Brașov, Romania; 3 Department of Medical and Surgical Specialties, Faculty of Medicine, University of Transilvania, 500036 Brașov, Romania; mariana.sporis@gmail.com (M.S.); daniela.mosoiu@hospice.ro (D.M.); 4 RMN Diagnostica, 500331 Brașov, Romania; 5 Faculty of Sociology, University of Transilvania, 500036 Brașov, Romania; flavia.hurducas@hospice.ro

**Keywords:** comfort care, aggressive interventions, final week of life, palliative care, advanced cancer, settings

## Abstract

*Background and Objectives*: End-of-life care in oncology demands a holistic, multidisciplinary framework that pivots clinical priorities from disease-directed therapies toward symptom optimization, comfort, and patient dignity. Within this context, the present study investigated care delivery patterns among advanced cancer patients during their final week of life. *Materials and Methods*: A retrospective medical record review was conducted, comparing an oncology ward, an intensive care unit, an inpatient palliative unit, and a home-based palliative care setting. We analyzed aggressive interventions, end-of-life care pathways, symptom management, medication use and rationalization, and transitions between care settings. *Results*: Among the 306 patients studied, diagnostic testing and artificial hydration were universally administered in the oncology and intensive care unit (*p* < 0.001), where prescriptions focused primarily on pain management. Conversely, palliative care settings addressed a wider range of terminal symptoms. The rationalization of non-essential medications took place between 1.8 and 2.9 days before death, varying significantly across medication classes and clinical settings (*p* < 0.001). Additionally, intensive care unit admissions were more prevalent among patients with advanced cancer, while home-based patients underwent the greatest number of care transitions. *Conclusions*: These findings highlight substantial differences in care across settings, emphasizing the need for coordinated planning, continuity, and patient-centered approaches to ensure comfort and dignity in the final week of life.

## 1. Introduction

The goal of palliative care at the end of life for patients with advanced cancer is to provide comfort care and to prevent suffering whenever possible [1]. Comfort care, especially in the final week of life for patients with cancer, is a multidimensional approach that focuses on alleviating suffering and promoting quality of life during the final stages of the illness. Defined broadly, comfort care aims to provide relief from distressing symptoms while respecting the patient’s wishes [2]. End-of-life comfort depends on several care factors, such as symptom severity, physical decline, emotional response, relationships, spiritual beliefs, personal values, and cultural background [3,4,5]. Moreover, focusing on comfort in the final week of life is associated with reduced rates of unnecessary hospital admissions and invasive interventions that may cause additional suffering without significant benefits [6].

In contrast, aggressive care often includes continued curative treatments or interventions focused on prolonging life. Often, patients with advanced cancer receive aggressive interventions, such as chemotherapy or hospital admissions shortly before death, which may affect their quality of life [7,8]. Studies show that late admission to a hospital correlates with a lack of timely transition to palliative care, resulting in significant patient distress and poor symptom control [7,9]. These misunderstandings regarding the goals of end-of-life care between patients with advanced cancer and providers can lead to a reliance on invasive interventions, rather than palliative measures [10].

In the dying phase, ethical dilemmas frequently arise at the intersection of patient autonomy and clinical decision making, generating moral distress among healthcare providers. These dilemmas commonly involve decisions regarding do-not-attempt resuscitation, artificial nutrition and hydration, palliative sedation, and withholding and withdrawing treatments [11]. Legal, social, cultural, and spiritual pressures also significantly shape the decision-making process [12]. Studies highlight the adverse effects of persisting with futile treatment, categorizing them into impacts on patients, their relatives, and healthcare professionals, as well as economic outcomes [12,13].

Patients with advanced cancer at end of life want to be comfortable and should be offered privacy and dignity [14]. Healthcare providers have to consider patients’ perspectives and preferences [15,16]. Navigating end-of-life care requires clinicians to align personalized management with patient and family goals while carefully weighing treatment burden against meaningful benefit in light of the patient’s prognosis [17].

In Romania, palliative care has developed progressively over the last three decades, largely pioneered by non-governmental organizations like Hospice Casa Sperantei, alongside expanding public sector initiatives. Despite these advances, national coverage remains heterogeneous, with marked disparities between acute hospital environments and specialized palliative care services. Specialized home-based palliative care operates primarily through non-governmental organizations to bridge structural gaps in the public health system. The service is delivered by interdisciplinary mobile teams comprising specialized palliative care physicians, nurses, social workers, and psychologists. Crucially, the Romanian healthcare system currently lacks a formal regulatory and legal framework for Advance Care Planning. Without a legal framework to document patient preferences in advance, clinical decision making is frequently deferred until the terminal phase, often leading to acute care crises and delayed transitions to comfort-centered care. Given this fragmented systemic backdrop and the resulting variability in decision making, evaluating clinical practices during the imminent dying phase is critical. Therefore, the aim of this study was to comparatively analyze management strategies for patients with advanced cancer in their final week of life across different care settings: an oncology ward, an intensive care unit, an inpatient palliative care unit, and a community home-based palliative care setting.

## 2. Materials and Methods

### 2.1. Design

This study employed a retrospective chart review methodology guided by the framework described by Gearing et al., which provides a systematic approach to medical record review research through six sequential steps: conception, literature review, research proposal, development of data abstraction tool, sample size determination, ethics approval, and study conduct [18]. Medical records were reviewed for patients with advanced cancer who died between January and September 2025 across different settings: an oncology ward, an intensive care unit, an inpatient palliative care unit, and a home-based palliative care service. The observation period chosen was the final week of life, to evaluate clinical interventions during the dying phase, where healthcare interventions and symptom management frequently peak.

### 2.2. Settings

The oncology ward and the intensive care unit were from Brasov County Emergency Hospital, a major regional healthcare facility in Romania. This hospital with 910 beds serves the Brasov region, managing a high-acuity caseload for an estimated population of over 600,000 residents. The facility acts as a critical intersection for diverse adult patients’ pathways, bridging the gap between high-density inner-city urban areas and remote rural communities. Its role is twofold: providing immediate life-saving interventions for acute trauma and emergencies, while also managing complex chronic progressions in specialized wards [19,20]. The inpatient palliative care unit and home-based services chosen operate as fully integrated care settings under Hospice Casa Sperantei (Brasov, Romania), the country’s largest non-governmental provider of specialized palliative care. This integrated model ensures seamless continuity of care across multiple modalities, including inpatient care, home-based services, outpatient clinics, and day-care centers for adult and pediatric populations [21,22,23].

### 2.3. Participants

We included patients over 18 years of age diagnosed with advanced cancer who died between January and September 2025 while undergoing care either in Hospice Casa Sperantei or in the Brasov County Emergency Hospital. Patients who died within the first 24 h of admission were excluded. The required sample size was determined using G*Power (version 3.1.9.7; Heinrich-Heine-Universität Düsseldorf, Düsseldorf, Germania) software [24], based on a 95% confidence level and a 5% margin of error, yielding a minimum target of 290 patients. To ensure the integrity of the findings, the final study included a total of 306 patients.

### 2.4. Data Collection

Data collection variables were operationalized based on the theoretical framework of an integrative review regarding patterns of care in the final week of life, specifically targeting clinical management, interventions, and care delivery during the imminent dying phase. For hospitalized patients treated in the oncology ward or intensive care unit, data were extracted directly from patients’ medical records. The home-based setting provided by Hospice Casa Sperantei is fully integrated with the inpatient palliative care unit, operating under unified clinical documentation. Both care settings utilize a comprehensive medical record-keeping system designed for holistic patient assessment, which incorporates validated symptom screening tools alongside standardized sections for documenting all interventions. Terminal patients receiving care at home were monitored daily by a specialized palliative care team, either through in-person home visits or structured telephone consultations, with all data systematically recorded in the medical records.

Grounded in a published integrative review framework [25], variables were categorized into two distinct patterns: comfort-centered care and non-comfort-centered care. Comfort-centered care included the use of end-of-life protocols, symptom management, rationalization of non-essential drugs, and routes of administration. Non-comfort-centered care captured aggressive interventions: diagnostic tests, artificial nutrition and hydration, antibiotics, radiological procedures, blood transfusions, chemotherapy, radiotherapy, cardiopulmonary resuscitation, intubation, and inter-setting transition during the final week of life.

### 2.5. Ethical Considerations

The study protocol was approved by the Ethics Committees of the Hospice Casa Sperantei (No 08/31.10. 2024) and County Emergency Hospital (No 10327/18.06.2025).

### 2.6. Data Analysis

Patient demographics and clinical characteristics were summarized using descriptive statistics. To determine statistically significant differences among the four care settings, we applied the Pearson Chi-square (χ^2^) test, utilizing Monte Carlo sampling for symptom presence, pharmacological symptom management, medication rationalization, and non-comfort-centered interventions. Monte Carlo sampling simulation was chosen specifically to ensure statistical validity, given the sample size imbalances across settings [26,27]. Kruskal–Wallis tests were conducted to evaluate differences in medication utilization across care settings during the final week of life. Cases with missing values were excluded listwise from all relevant analyses. A *p*-value of <0.05 was considered statistically significant. Data collection and statistical analysis were conducted using IBM SPSS Statistics for Windows, Version 27.0 (IBM Corp., Armonk, NY, USA).

## 3. Results

During the study period, 306 patients meeting the inclusion criteria were analyzed. As presented in Table 1, over half of the cohort died in a home-based setting (n = 161, 52.6%), followed by the inpatient palliative care unit (n = 89, 29.1%), the oncology ward (n = 49, 16.0%), and the intensive care unit (n = 7, 2.3%). The median age of the whole sample was 70.29 (19–99) years and 50.2% were male. The predominant primary cancer sites were digestive (35.0%), lung (16.1%), genitourinary (16.0%), head and neck (9.5%), and breast (9.5%), followed by less frequent primary origins including bladder, skin, kidney, bone, and hematological malignancies. High clinical complexity was evident across the cohort, with over 77% of patients presenting with metastatic disease and more than 75% having documented comorbidities.

### 3.1. Use of Aggressive Interventions

The frequency of clinical interventions varied significantly across care settings (Table 2). In oncology, diagnostic testing and artificial hydration were universally administered (100%), while antibiotic therapy and resuscitation occurred in 69% and 37% of patients, respectively. The intensive care unit exhibited the highest treatment intensity, with all patients receiving diagnostic testing, artificial hydration, and resuscitation, alongside high rates of antibiotic administration (86%) and intubation (71%). Conversely, specialized palliative care settings demonstrated a substantially lower prevalence of invasive procedures. Within the inpatient palliative care unit, diagnostic testing, antibiotic administration, and artificial hydration predominated, whereas home-based care recorded the lowest overall frequency of aggressive interventions. Oral chemotherapy was continued in only two patients: one in the inpatient unit and one receiving home-based care. Minor surgical interventions occurred in just three cases, all limited to colostomy and gastrostomy management; these comprised one inpatient who returned to the inpatient unit post-procedure, and two home-based patients requiring brief hospital admissions for stoma troubleshooting before returning home.

### 3.2. Medication Rationalization

Table 3 presents the comparative analysis of the total number of active medications administered per patient across the four care settings at two distinct timepoints: 7 days prior to death and on the day of death. Statistically significant differences in medication count were observed among settings both at 7 days prior to death (χ^2^ = 56.08, *p* < 0.001) and on the day of death (χ^2^ = 98.40, *p* < 0.001). At 7 days prior to death, patients in acute care settings received a significantly higher number of medications compared to those in palliative care settings. The highest median medication counts were recorded in the intensive care unit [Median: 11 (IQR: 9–14)] and the oncology ward [Median: 10 (IQR: 8–12)]. In contrast, significantly lower baseline medication counts were documented in the inpatient unit [Median: 6 (IQR: 4–8)] and in home-based care [Median: 4 (IQR: 3–6)]. On the day of death, these disparities widened further. Active deprescribing in the inpatient palliative unit reduced median medications from 6 to 4 [IQR: 3–6], aligning with home-based care [Median: 4 (IQR: 3–6)] and reflecting intentional medication rationalization during the final hours of life. Conversely, high pharmacological burden persisted in oncology [Median: 10 (IQR: 8–12)] and escalated in the intensive care unit [Median: 12 (IQR: 10–14)].

Deprescribing of non-essential medications differed significantly across care settings (*p* < 0.001), encompassing vitamins, cardiovascular agents, anticoagulants, and gastric protectors (Table 4). The timeline for medication rationalization varied by drug class: antidiabetic agents were discontinued earliest (mean 2.9 days prior to death), whereas gastric protectors persisted longest, being rationalized a mean of 1.8 days before death.

### 3.3. Routes of Medications Administration

Oral and subcutaneous routes predominated in both the inpatient unit and home-based setting, providing non-invasive options for symptom management. Conversely, the oncology ward and intensive care unit demonstrated a significantly higher reliance on intravenous administration (*p* < 0.001).

### 3.4. Patient Transitions in the Final Week of Life

Over 40% of patients underwent at least one transition across care environments in their final week of life (*p* < 0.001). Relocations originated primarily from home-based setting (n = 93/161), with 61 patients admitted to the inpatient palliative care unit and 32 admitted to acute hospital services due to severe dyspnea, bleeding, bowel obstruction, stroke, or delirium. Notably, some patients experienced two hospital admissions during their final week of life, most of them being from home-based setting. Within acute care settings, transfers from oncology to intensive care unit (n = 10) occurred more frequently than transfers to the inpatient palliative care unit (n = 6).

### 3.5. Using Care Pathways

End-of-life care pathways were implemented exclusively within palliative care settings, with adoption rates substantially higher in the inpatient unit than in the home-based setting. Notably, clinical recognition of the terminal phase did not consistently translate into pathway activation, suggesting persistent procedural barriers to formal protocol initiation despite timely prognosis. Across all activated pathways, the mean duration of care was 3.28 days (range: 6 h to 27 days).

### 3.6. Symptom Management According to Care Pathways

Symptom management varied significantly across care settings (*p* < 0.001), with specialized palliative care settings demonstrating the highest degree of pathway alignment. Within dedicated palliative care settings, 43% of patients received four key medication classes (analgesics, antiemetics, psychotropics, and anticholinergics) while 31% received three. Conversely, prescribing patterns in the oncology ward and intensive care unit were markedly narrower, with patients receiving no more than one or two medication classes.

Patterns of care during the final week of life varied substantially across clinical settings (Table 5). Marked disparities were observed in the frequency of aggressive interventions, pharmacotherapeutic strategies, and adherence to end-of-life protocols, underscoring the profound influence of the care environment on terminal patient trajectories.

## 4. Discussion

In this retrospective medical record review, end-of-life care patterns among patients with advanced cancer during their final week of life were evaluated across different settings: oncology ward, intensive care unit, inpatient palliative care unit, and home-based palliative care setting. The home-based setting was found to offer the least aggressive approach, characterized by minimal procedural interventions, due to both palliative care philosophy and structural limitations on hospital-based treatments. Similarly, the inpatient palliative care unit prioritized comfort and symptom control, while systematically avoiding non-beneficial treatments. Conversely, patients managed in the oncology ward or the intensive care unit experienced substantially higher rates of aggressive interventions, including routine diagnostic tests, artificial hydration and resuscitation.

Comfort-centered care in palliative care settings reflects a strong ethical commitment to patient dignity [28]. The findings create a distinct dichotomy between comfort- and non-comfort-centered care. In both the intensive care unit and the oncology ward, intervention intensity remains disproportionately high despite a recognized terminal prognosis. This phenomenon is frequently attributed to “therapeutic obstinacy”, where an institutional culture oriented toward curative-intent care, combined with the continuous availability of life-sustaining technology, prioritizes physiological stabilization over quality of dying [29,30]. Our findings align with international evidence suggesting that dedicated palliative care settings effectively attenuate intervention intensity at the end of life [31,32]. The observed clinical practice variations across settings raise fundamental ethical questions regarding the appropriateness and proportionality of care during the dying phase. Sustained treatment intensity during this period compromises the bioethical principle of non-maleficence, shifting care toward therapeutic obstinacy and exposing patients to interventions whose potential burdens exceed any plausible therapeutic benefit.

A substantial portion of patients in home-based care require hospital admission in their final week of life, pointing to the fragility of home-based care. These late-stage hospitalizations are associated with ambulance transfers and a significant barrier to a good death [33,34]. Transforming the home, a preferred end-of-life environment for care [35,36], into a site of clinical crisis, directly undermines patient tranquility and dignity.

The rationalization of non-essential medications at the end of life is not merely a routine clinical adjustment, but a profound ethical imperative [37] that restores the proportionality of care, aligning medical actions with the true needs of the dying patient. Although deprescribing medications is aligned with comfort [38,39] this study shows a process of rationalization only in palliative care settings. Despite clinical guidelines explicitly recommending deprescribing near end of life [40,41,42], the existing literature consistently confirms a widespread failure to rationalize non-essential pharmacotherapy in clinical practice [43,44,45].

The high prevalence of aggressive medical interventions in the final week of life identified in this study reflects persistent structural difficulties within healthcare systems regarding the early recognition of the dying phase and lack of advance directives. Evidence highlights that advance care planning and early goal of care clarification successfully shift the terminal care pathway away from aggressive intervention toward dignity and comfort [46,47,48]. Despite the absence of statutory advance directive legislation in Romania, specialized palliative care teams routinely conduct informal goals-of-care discussions to establish comfort-centered trajectories before the final week of life. Conversely, oncology wards and intensive care units typically operate without structured care planning protocols, fostering reflexive medicalization near death. In these acute care environments, clinicians, trained predominantly to prolong survival, frequently misinterpret the cessation of disease-directed treatment as professional failure or patient abandonment. Consequently, aggressive interventions are often initiated not solely for physiological benefit, but as defensive medicine driven by surrogate pressure and fear of medicolegal recourse.

Several limitations must be acknowledged. Firstly, the retrospective medical record review resulted in an unequal distribution of patients across the four settings, which may affect the generalizability of the findings and introduce selection bias when comparing inter-site outcomes. Secondly, while the quantitative approach identifies aggressive intervention and failure to rationalize non-essential medications and frequent transitions, it fails to capture the underlying “why”. Thirdly, a significant gap exists regarding the psychosocial and spiritual dimensions of care due to inconsistent documentation practices. In the intensive care unit and the oncology ward, medical records lacked dedicated sections for these fields, making it difficult to extract comparable holistic data. This absence not only limits the analysis of comfort-centered care, but also exposes a systemic under-documentation of the non-medical needs of dying patients, needs that are essential for truly comprehensive, patient-centered treatment planning.

This study has several strengths. First, the data collection framework was rigorously informed by a comprehensive literature review, ensuring the inclusion of validated clinical indicators for the final week of life care. Second, the four-setting comparative design allows for a unique analysis of how institutional culture influences medical intensity, providing a broader systemic perspective than single-site evaluations. Finally, by specifically targeting the last seven days of life, this study captures the critical period where ethical decisions regarding medical proportionality and the transition to comfort-oriented care are most impactful, offering high-resolution insights into the realities of the dying trajectory across different places of care.

To enhance terminal care, hospitals should implement standardized end-of-life pathways and systematic deprescribing protocols across all departments, extending specialized palliative expertise into oncology wards and intensive care units. Essential to this framework are proactive goals-of-care discussions, which enable patients and families to establish care preferences prior to acute crises.

Future research should prioritize qualitative studies to investigate why clinicians persist with aggressive interventions despite poor prognoses, while also addressing documentation gaps related to the underrepresented social and spiritual dimensions of care. Expanding into multicenter studies will ensure these findings apply across diverse clinical and demographic settings. Additionally, future investigations should examine the clinical and ethical implications of managing aggressive medical interventions in patients with end-stage non-oncological chronic diseases whose therapeutic options are exhausted. This includes evaluating decision-making processes surrounding the continuation or limitation of burdensome procedures at the end of life, aiming to prevent unnecessary invasive treatments and improve comfort and dignity for patients and their families.

## 5. Conclusions

This study demonstrates that the management of patients in their final week of life is significantly influenced by the healthcare setting, creating a lottery of location that raises profound ethical concerns. The quantitative data reveal a stark contrast between the therapeutic obstinacy found in the intensive care unit and the oncology ward, characterized by sustained medical intensity, with comfort-centered care prevalent in palliative care settings. Furthermore, the shift from community care to hospitalization underscores the fragility of home-based care, where the failure to manage crisis symptoms transforms preferred environments into sites of clinical distress.

Ultimately, the quality of a death should not be contingent upon the ward in which a patient is admitted. To align clinical practice with the ethical principles of non-maleficence and dignity, healthcare systems must harmonize end-of-life protocols across all settings. The shift from aggressive curative efforts to comfort care is more than a change in protocol; it is a fundamental commitment to ensuring that the patient’s final days are governed by proportionality and deep respect for their individual journey.

## Figures and Tables

**Table 1:** Sociodemographic and clinical characteristics of patients across care settings.

Category	All	Inpatient Unit	Home-Based	Oncology	Intensive Care Unit	*p* Value
**Number of patients**	306 (%)	89	161	49	7	
**Age** (average, min, max)	70.29	71.53 (39–89)	71.15 (19–99)	65.9(43–80)	65.5 (53–74)	0.005
**Gender**						
Male	154 (50.2)	46	76	27	5	0.529
Female	152 (49.8)	43	85	22	2	
**Diagnosis**						
Digestive	107 (35)	32	59	13	3	
Lung	49 (16.1)	13	23	12	1	
Head & Neck	40 (9.5)	5	27	7	2	
Female genital tumors	30 (9.8)	7	14	8	1	
Breast	29 (9.5)	11	14	4	0	
Male genital tumors	19 (6.2)	6	12	1	0	
Bladder	10 (3.2)	7	3	0	0	
Kidney	7 (2.3)	3	1	3	0	
Hematologic	7 (2.3)	4	3	0	0	
Melanoma	6 (1.9)	1	4	1	0	
Bone	1 (0.3)	0	1	0	0	
**Comorbidities**						0.920
Yes	229 (74.8)	68	118	38	5	
No	76 (25.2)	21	42	11	2	
**Metastases**						0.124
Yes	237 (77.7)	67	120	44	6	
No	68 (22.3)	22	41	4	1	
**Symptoms**						
Pain	295 (97%)	88	156	49	7	0.191
Fatigue	247 (81%)	78	129	35	5	0.118
Lack of appetite	228 (75%)	70	120	35	3	0.192
Constipation	191 (63%)	52	105	32	2	0.480
Dysphagia	166 (54%)	54	91	17	4	0.016
Xerostomia	117 (38%)	39	64	14	0	0.195
Nausea	113 (37%)	33	54	25	1	0.102
Drowsiness	90 (30%)	25	45	19	1	0.446
Breathlessness	85 (28%)	29	35	18	3	0.067
Delirium	70 (23%)	28	33	8	1	0.140
Noisy breathing	57 (19%)	21	30	4	2	0.990
Bleeding	49 (16%)	15	24	10	0	0.748

**Table 2:** Use of non-comfort-oriented care interventions across care settings.

Non-Comfort-Oriented Interventions	Inpatient Unit N-89	Home-Based N-161	Oncology N-49	Intensive Care Unit N-7	*p* Value *
Diagnostic tests	24	3	49	7	0.001
Radiological procedures	4	1	20	1	0.001
Use of antibiotics	10	9	33	5	0.001
Artificial nutrition	4	0	13	2	0.001
Blood transfusion	0	1	8	0	0.001
Chemotherapy	1	1	5	0	0.010
Radiotherapy	0	0	1	0	0.198
Intubation	0	0	0	5	0.001
Surgery	1	2	0	0	0.838
Resuscitation	0	0	18	7	0.001
Artificial hydration	8	3	49	7	0.001

* Differences in using non-comfort-oriented interventions between care settings were tested using Chi-square (χ^2^) test with Monte Carlo simulation.

**Table 3:** Comparison of active medication counts across care settings at 7 days prior to death and on the day of death.

Setting	Patients (No.)	Median (IQR) 7 Days Before Death	Mean Rank 7 Days Before Death	Median (IQR) Day of Death	Mean Rank Day of Death
**Home-Based**	161	4 (3–6)	114.45	4 (3–6)	117.03
**Inpatient Unit**	89	6 (4–8)	145.98	4 (3–6)	117.31
**Oncology**	49	10 (8–12)	212.14	10 (8–12)	234.52
**Intensive Care Unit**	7	11 (9–14)	210.33	12 (10–14)	248.58
Kruskal–Wallis test			χ^2^ = 56.08, df = 3, *p* < 0.001		χ^2^ = 98.40, df = 3, *p* < 0.001

**Table 4:** Rationalization of medication across care settings.

Rationalization of Medication	Patients (No.)	Inpatient Unit	Home-Based	Oncology	Intensive Care Unit	*p* Value *
**Vitamins**	**67**					0.001
Yes	27	11	4	12	0	
No	40	6	14	17	3	
**Heart Medication**	**112**					0.001
Yes	68	34	31	3	0	
No	44	7	19	14	4	
**Corticosteroids**	**147**					0.001
Yes	59	23	32	4	0	
No	88	14	41	29	4	
**Anticoagulants**	**57**					0.001
Yes	23	15	3	5	0	
No	34	3	7	20	4	
**Gastric protector**	**210**					0.001
Yes	112	54	55	3	0	
No	98	11	45	39	3	
**Antibiotics**	**69**					0.001
Yes	12	4	5	3	0	
No	57	10	9	33	5	
**Liver protector**	**46**					0.001
Yes	16	14	1	1	0	
No	30	0	4	24	2	
**Diuretics**	**111**					0.001
Yes	57	33	19	5	0	
No	54	9	15	25	6	
**Laxatives**	**162**					0.001
Yes	85	40	43	2	0	
No	77	12	46	17	2	
**Diabetes medication**	28					0.18
Yes	17	10	7	0	0	
No	11	4	6	1	0	

* Differences between care settings were tested using Chi-square (χ^2^) test with Monte Carlo simulation.

**Table 5:** Summary of care patterns in the final week of life across settings.

Setting Interventions	Inpatient Unit	Home-Based	Oncology	Intensive Care Unit
**The most used non-comfort-oriented ** **care interventions**				
Diagnostic tests	++	+	++++	++++
Use of antibiotics	+	+	+++	++++
Artificial hydration	+	+	++++	++++
Resuscitation	-	-	++	++++
**Medication**				
**Average final-day medications**	4.1	4.5	10.2	13
**Administration route**				
Subcutaneous	++++	++++	+	-
Intravenous	+	-	+++	++++
**Using end-of-life care pathways**	++++	+++	-	-
**Patient transition between places of care**				
Admission to acute care or intensive care unit	-	+	++	N/A
Transfer to inpatient palliative care unit	N/A	++	+	-
Terminal discharge to home-based	+	N/A	-	-

“+” fewer than 25% of patients received intervention; “++” 26–50% of patients received intervention; “+++” 51–75% of patients received intervention; “++++” over 75% of patients received intervention; “-” the intervention did not occur; “N/A” not applicable to that setting.

## Data Availability

The data presented in this study are available on request from the corresponding author. The data are not publicly available due to privacy restrictions and institutional data protection policies.
